# Clinical and CT characteristics of human metapneumovirus-associated severe pneumonia in children in Beijing

**DOI:** 10.1186/s13052-025-01973-1

**Published:** 2025-05-09

**Authors:** Weihan Xu, Xiaoyan Zhang, Yuhong Guan, Ruxuan He, Xiang Zhang, Jinrong Liu

**Affiliations:** Department of Respiratory Medicine, Beijing Children’s Hospital, National Center for Children’s Health, China National Clinical Research Center of Respiratory Disease, Capital Medical University, NO.56, Nanlishi Road, Beijing, 100045 P.R. China

**Keywords:** Human metapneumovirus, Pneumonia, Children, Computed tomography

## Abstract

**Background:**

Human metapneumovirus (HMPV) has been increasingly appreciated as a cause of lower respiratory tract infection among children. The purpose of this paper is to determine the radiographic and clinical features of children with HMPV lower respiratory disease.

**Case Presentation:**

We reviewed seven pediatric patients with severe pneumonia due to HMPV admitted to the Department of Respiratory Medicine, Beijing Children’s Hospital were assessed in our study from January to July 2023. Unlike other common viral, lobar or segmental consolidation, air bronchograms, and bronchial wall thickening were the most commonly observed HRCT findings in HMPV-associated pneumonia. C-reactive protein (CRP) levels, ranged 35 mg/L to 146 mg/L, and the median WBC count were significantly increased in children with HMPV-associated pneumonia than the normal level. Two patients were co-detected with Haemophilus influenzae and streptococcus pneumoniae, respectively. Five patients were treated with empirical antibiotics prior to the bacterial test results.

**Conclusions:**

Some pediatric HMPV-associated pneumonias were characterized by lobar or segmental consolidation in CT and the significantly elevated CRP levels, which may mimic Mycoplasma Pneumoniae or bacterial infection. Healthcare providers should consider HMPV as a possible causative pathogen, perform laboratory tests for prompt diagnosis, and limit unnecessary antibiotic treatment.

## Background

Identified for the first time in 2001, a newly virus from the Paramyxoviridae family, human metapneumovirus (HMPV) has emerged as a significant viral pathogen causing acute upper and lower respiratory tract infections in children, the elderly and immuno-compromised patients [1]. HMPV has been reported to exhibit a wide variation of symptoms from cough to bronchiolitis and pneumonia requiring hospitalization, which may be similar to those of respiratory syncytial virus (RSV) [2]. HMPV shows seasonal variation, as an epidemic virus that occurs in outbreaks, with the majority of infections being observed in late winter through spring. Annual rates of hospitalization associated with HMPV in were approximately 1 to 1.2 per 1000 children under 5 years old in the United State, and even higher rates in European children [3]. Hospitalized children under 5 years with HMPV infection were more likely to require supplemental oxygen and had longer pediatric intensive care unit stays than their counterparts without HMPV infection [4]. Recently, accumulating studies have indicated the radiologic features of HMPV infection [5–7]. However, few reports have systematically characterized the CT features of HMPV-associated pneumonia in children.

Between 1 January and 31 July 2023, there was an outbreak of influenza-like illness in Beijing, which HMPV was a primary pathogen identified in children. Many children with HMPV infection presented with pneumonia who are hospitalized. In contrast to recent studies, characterized by bronchial wall thickening, ground-glass opacities (GGO), and centrilobular nodules, our case series of HMPV-associated pneumonia demonstrated different CT and clinical features [6, 7]. To address this issue, strengthening case finding, we assessed the characteristic chest radiographic and clinical features of children with laboratory-confirmed HMPV who suffered severe lower respiratory disease in this outbreak.

## Cases presentation

Seven HMPV-positive (RT-PCR positive) children with severe pneumonia hospitalized in the Department of Respiratory Medicine at Beijing Children’s Hospital Between 1January and 31 July 2023 were assessed in our study. Severe pneumonia was defined according to the 2013 WHO definition: cough and/or increased work of breathing and significant tachypnea (respiratory rate > 70 breaths per minute in infants and > 50 breaths per minute in older children) with any 1 of severe respiratory distress, oxygen saturation < 90%, cyanosis, refusal to eat, dehydration, disturbance of consciousness, and convulsions [8]. Nasopharyngeal swab or bronchoalveolar lavage (BAL) were tested for the detection of HMPV, adenovirus, corona-viruses, human rhinovirus, influenza A/B viruses, parainfluenza viruses, RSV, Chlamydophila pneumoniae, bocavirus, and *Mycoplasma Pneumoniae* by a RT-PCR assay. Sputum and/or BAL for bacterial culture were performed for all seven patients, and BAL for next-generation sequencing were performed for four patients. Children identified as HMPV-positive in this report had HRCT obtained as part of their routine clinical care. The HRCT results were reviewed independently by two pediatric radiologists with 20 years and 5 years of experience in thoracic imaging. The decisions for BAL procedures and laboratory testing were clinician directed, as routine clinical care, and laboratory results were assessed retrospectively.

Seven children with a diagnosis of HMPV-associated severe pneumonia were enrolled in our case series. Their ages ranged from 7 months to 7 years 5 months old. All patients had cough and fever, four patients also had wheezing and/or tachypnea, one patient also had blood-stained sputum. All patients developed pneumonia as evident on CT. For five of seven (71.4%) patients, pneumonia was diagnosed within 5 days after the onset of symptoms. In total, three patients had a history of atopic dermatitis, two patients had a history of allergic rhinitis (Table 1).

**Table 1:** Demographic and clinical features of 7 children with human metapneumovirus-associated severe pneumonia. Abbreviations: WBC, white blood cell; CRP, C-reactive protein; LDH, lactase dehydrogenase; GGO, ground-glass opacities

Age Gender	Clinical presentation	HRCT findings	Laboratory findings						Personal Atopic disease	Co-infection	Treatment		
			WBC ×10^9^/L	CRP mg/L	Neu %	D dimer ng/mL	LDH IU/L	IgE IU/L			Non-invasive	Glucocorticoid	Bronchoscopy
7yrs 5mo F	Cough, fever, wheezing, tachypnea	Bilateral, consolidation, bronchial wall thickening, GGO	17.14	78	93.2	0.22	302	128	NO	NO	Undone	2 mg/kg	Hyperemic edematous mucosa, diffuse mucus secretion, mucus plug
3yrs 8mo F	Cough, fever, blood-stained sputum	Unilateral, GGO, consolidation, centrilobular nodules, bronchial wall thickening	14.64	114	84.3	0.34	189	177	NO	NO	Undone	2 mg/kg	Hyperemic edematous mucosa, white viscous secretions, mucus plug, mucosa erosion, partially occluded lumens
7mo M	Cough, fever, wheezing, tachypnea	Unilateral, GGO, consolidation, bronchial wall thickening	7.76	59	63.3	0.26	301	31	NO	Haemophilus influenzae	Done	2 mg/kg	Mucosal hyperaemia, diffuse viscous secretions
3yrs 6mo M	Fever, cough, wheezing	Unilateral, GGO, consolidation, bronchial wall thickening	16.07	35	76.3	0.31	248	527	Allergic rhinitis	streptococcus pneumoniae	Undone	2 mg/kg	Hyperemic edematous mucosa, white viscous secretions
1yrs 11mo F	Cough, fever, tachypnea	Bilateral, consolidation, centrilobular nodules, bronchial wall thickening	15.02	75	77	0.72	496	45	NO	NO	Done	2 mg/kg	Hyperemic edematous mucosa, white viscous secretions
4yrs 4mo M	Cough, fever, diarrhea	Bilateral, consolidation, centrilobular nodules, GGO, bronchial wall thickening, pleural effusion	4.27	79	52.1	0.32	277	145	Allergic rhinitis	NO	Undone	2 mg/kg	Hyperemic edematous mucosa, mucosa erosion, diffuse viscous secretions, mucus plug
6yrs 2mo F	Cough, fever	Bilateral, GGO, consolidation, centrilobular nodules, pleural effusion	7.64	146	82.8	0.46	367	119	Atopic dermatitis	NO	Undone	4 mg/kg	Hyperemic edematous mucosa, mucosa erosion, white viscous secretions

Blood tests were performed in all seven patients. Interestingly, C-reactive protein (CRP) levels, ranged 35 mg/L to 146 mg/L, were significantly increased in children with HMPV-associated pneumonia than the normal level. Moreover, four patients had a white blood cell count > 10 × 10^9^/L, while three patients had a normal white blood cell count. The median WBC count was 14.64 > 10 × 10^9^/L and N was 77% in patients with HMPV-associated pneumonia. Meanwhile, most patients had elevated levels of lactase dehydrogenase (LDH), D-dimer, and IgE antibodies at the time of admission. Of the seven patients, five (71.4%) of them were identified only with HMPV, while two (28.6%) patients were co-detected with Haemophilus influenzae and streptococcus pneumoniae, respectively (Table 1).

**Fig. 1:**
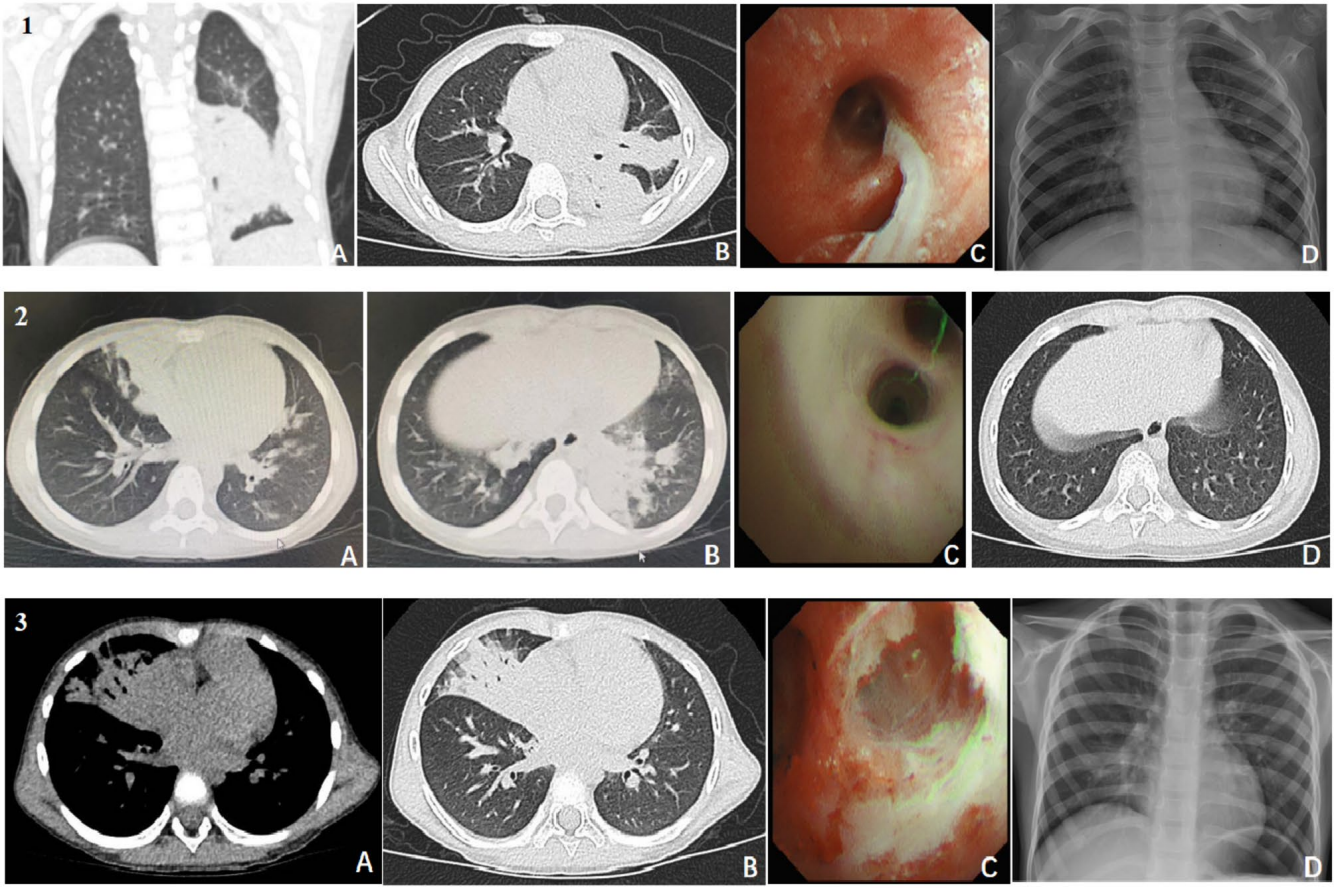
**1.** Pneumonia due to human metapneumovirus. Pneumonia due to HMPV in 3-year-old children who presented with fever, cough, and blood-stained sputum. (**A**) Axial high-resolution (0.625-mm collimation) chest CT images showed a segmental consolidation in the right upper lobe. The air bronchogram was well delineated within the consolidation. (**B**) Lung window image showed patchy centrilobular nodules around the segmental consolidation. In addition, bronchial wall thickening was seen in the right lower lobe. (**C**) Bronchoscopy showed a partially occluded lumen in segmental bronchi. (**D**) Anteroposterior chest radiograph showed disappeared air-space consolidations after 1 week of treatment. **2.** Pneumonia due to HMPV in 4-year-old children who presented with fever and cough. (**A**) Coronally reconstructed CT image demonstrates lobar consolidation in the left lower lobe. (**B**) Axial high-resolution (1.25-mm collimation) chest CT images shows patchy GGO, centrilobular nodules and lobar consolidation. (**C**) Bronchoscopy showed the sputum plugs in the bronchial cavity of the lower left and the posterior branch. (**D**) Anteroposterior chest radiograph showed disappeared air-space consolidations after 1 week of treatment. **3.** Pneumonia due to HMPV in 4-year-old children who presented with fever and cough. **A** and **B**) Axial high-resolution (1.25-mm collimation) chest CT images show patchy GGO, centrilobular nodules around the segmental consolidation. **C**) Bronchoscopy showed mucosa erosion and white viscous secretions in the left lower posterior basal branch. **D**) HRCT showed disappeared consolidations after 9 days of treatment

Upon admission, all seven children underwent bronchoscopy and bronchoalveolar lavage. The most frequent bronchoscopy findings were mucosal hyperaemia and oedema (*n* = 7), white viscous secretions (*n* = 4), diffuse mucus secretions (*n* = 3), and mucosa erosion (*n* = 3). The mucus plugs were seen in the bronchial cavity of the lungs that blocked the lumen in three children, and the lumen was partially occluded after lavage in one child (Figs. 1; Table 1).

The chest CT was performed among all seven patients, four of these patients had bilateral involvement. Strikingly, the most frequent CT finding of HMPV-associated pneumonia was a lobar and/or segmental air-space consolidation. Air-space consolidation was observed in all patients, which was single (*n* = 5), two distinct areas (*n* = 2) (Figs. 1). The distributions of pulmonary consolidation were lobar (*n* = 3) or segmental (*n* = 4). Air bronchograms were observed within areas of consolidation (*n* = 5) and bronchial wall thickening (*n* = 6) were common findings. Although GGO was observed in six patients, it was patchy and minimal in extent in four of them, usually observed around consolidation. Small areas of centrilobular nodules were observed in four patients, also surrounding the lobar or segmental consolidation. In six patients with follow-up CT/chest X-ray, the previously observed air-space consolidations were disappeared (Figs. 1; Table 1).

Since the CRP levels and WBC count were significantly elevated, regardless of the bacterial test results, five pneumonia patients were treated with empirical antibiotics prior to admission, seven patients were treated with empirical antibiotics during hospitalization. Of these, two patients required in intravenous administration of cephalosporin based on the results of bacteria culture and drug sensitivity (Table 2). Intravenous drip of methylprednisolone (2-4 mg/kg.d) was given to all seven patients with HMPV-associated pneumonia. Three children required supplemental oxygen, and two children with serious respiratory failure required non-invasive ventilation. (Table 1).

**Table 2:** Demographic features and outcomes of the patients

Variable	No. (%)
Symptom duration prior to admission, days, median	5 (4, 20)
Duration of fever, days, median	5 (3, 16)
Antibiotics prior to admission	5 (71.4%)
Azithromycin	3 (42.9%)
Cephalosporins	3 (42.9%)
Antibiotics during hospitalization	6 (85.7%)
Azithromycin	3 (42.9%)
Cephalosporins	4 (51.7%)
Oxygen requirement	3 (42.9%)
Nasal/mask oxygen	3 (42.9%)
Noninvasive ventilation	2 (28.6%)
Mechanic ventilation	0
ICU transfer	0
Length of stay among hospitalization, days, median	6 (5, 9)
PPD positive	0
Positive specific IgE	4 (57.1%)

Values are No. (%) or the median (range) unless otherwise indicated

## Discussion and conclusions

Our study demonstrated the clinical and radiologic findings of children with HMPV-associated pneumonia. The most interesting finding was the exhibition of lobar or segmental consolidation, which is generally expected to be observed in severe *Mycoplasma Pneumoniae* pneumonia or bacterial infection, accompanied by a significant elevated level of CRP.

HMPV has a propensity to infect ciliated airway epithelial cells, it is easily justifiable for airway centric pattern to be common radiological feature in HMPV-associated pneumonia [9]. Previous studies have revealed that HMPV is most likely to manifest as airway centric disease presenting as bronchitis or bronchiolitis, characterized by peri-bronchial ground-glass opacities, bronchial wall thickening, ill-defined centrilobular nodules, which is similar to other paramyxoviridae (parainfluenz3a virus and respiratory syncytial virus) [6, 10]. To our best knowledge, this is the first report showed that HMPV infection can present a lobar or segmental consolidation on chest CT examination in some children. Although we could not exactly explain the underlying mechanism the different radiographic presenting of HMPV, we assume some kinds of different host immune responses to the same pathogen in pediatric patients. It is possible that child’s less mature immune responses may allow HMPV to spread into the peripheral bronchiole and lung parenchyma, lead to extensive air space exudation. Therefore, mucosal hyperaemia and oedema, mucus secretions, even mucus plugs were observed under bronchoscopy.

It has been shown that IL-6, TNF-α, and IL-18 levels were found to be significantly higher in children with HMPV pneumonia, indicating the role of inflammation in HMPV-mediated pneumonia [11]. Persistent high fever and progressive hypoxia suggested a cytokine storm. Patients who have acute onset respiratory distress, such as dyspnea with or without wheezing, should be treated as soon as possible with an early and adequate dosage of systemic immune modulators (corticosteroids and/or IVIG), which may reduce aberrant immune responses in the potential stage of acute respiratory distress syndrome. In line with these studies, after being treated with methylprednisolone in all patients, their conditions significantly improved in our report.

Moreover, children with HMPV pneumonia had significantly increased CRP levels, and were more likely to have a WBC > 10 × 10^9^/L in our report, which was generally expected to be bacterial infection [12]. Therefore, concomitant bacterial infection was assessed based on relevant cultures and/or next-generation sequencing. We only detected bacterial co-infection in two cases, and no *Mycoplasma Pneumoniae* co-infection case when we checked their laboratory and microbiological tests. Since the elevated CRP level and CT finding similar to *Mycoplasma Pneumoniae* and bacterial pneumonia, most patients were treated with empirical antibiotics prior to the bacterial test results. Despite the fact that there was less likely to have co-infection, the empirical antibiotic prescription rate was still high.

In summary, we report clinical and radiologic features for children with HMPV-associated pneumonia. Lobar or segmental consolidation in CT, which may mimic *Mycoplasma Pneumoniae* or bacterial infection, was manifested in all seven children. When confronted with pediatric pneumonia patients that exhibit these features demonstrated, we should consider HMPV as a possible causative pathogen, and perform laboratory tests for prompt diagnosis.

## Data Availability

The data used and analyzed during the current study are available from the corresponding author on reasonable request.
